# Biomimetic Chitosan/Polyvinyl Alcohol–Glycerol Scaffolds Inspired by Porcupine Quills for Segmental Bone Defect Repair

**DOI:** 10.3390/jfb17040177

**Published:** 2026-04-03

**Authors:** Jingwen Yang, Zihao Zhao, Zengtao Song, Lei Cao, Xifan Mei, Xing Zhang

**Affiliations:** 1 Institute of Metal Research, Chinese Academy of Sciences, Shenyang 110016, China; 2 School of Materials Science and Engineering, University of Science and Technology of China, Shenyang 110016, China; 3 Liaoning Provincial Key Laboratory of Medical Tissue Engineering, Jinzhou 121000, China; 4 Liaoning Provincial Collaborative Innovation Center of Medical Testing and Drug Development, Jinzhou 121000, China; 5 Liaoning Provincial Collaborative Innovation Center for Health Promotion of Children and Adolescents of Jinzhou Medical University, Jinzhou 121000, China

**Keywords:** bone regeneration, biomimetic material, porcupine quill structure, angiogenesis

## Abstract

Segmental bone defects are large, non-healing injuries characterized by insufficient structural support and limited bioactivity, posing a significant clinical challenge. In this study, we developed biomimetic chitosan/polyvinyl alcohol–glycerol (CS/PG) scaffolds inspired by porcupine quills, which were fabricated via fused deposition modeling and unidirectional freeze casting. The as-prepared scaffold featured a dense outer layer of polyvinyl alcohol–glycerol (PG) with high compressive strength (24.21 ± 0.11 MPa at 25% strain) and an oriented inner foam of chitosan (CS). The CS foam was further incorporated with poly (3,4-ethylenedioxythiophene) polystyrene sulfonic acid (PEDOT:PSS, denoted as PP) and amorphous zinc phosphate (AZP) to form PP-AZP-CS/PG, aimed at enhancing neural conductivity and stimulating blood vessel formation, respectively. The in vitro results indicated that the biomimetic scaffolds exhibited excellent biocompatibility while significantly enhancing angiogenesis and osteogenesis capabilities. In a rabbit radial segmental defect model, PP-AZP-CS/PG achieved robust bone regeneration, attaining a bone volume/total volume of approximately 26.22% after implantation for 8 weeks. Overall, this biomimetic scaffold demonstrated that integrating hierarchical design with additional bioactive components enhanced mechanical support while promoting new bone regeneration, addressing critical challenges in segmental bone defect repair.

## 1. Introduction

Segmental bone defects, defined as large bone injuries resulting in the fragmentation or absence of a segmented bone, remain a critical challenge due to the inability of bone to self-heal with such extensive damage [1,2,3]. Untreated segmental bone defects can lead to nonunion (persistent failure of bone bridging) and the loss of mechanical support due to a lack of vascular supply and neural regeneration at the injury area [4,5,6,7,8]. Current scaffolds with good mechanical strength commonly provide structural stabilization yet insufficient bioactivity, resulting in poor tissue ingrowth, necrosis in the defect core, and incomplete regeneration [9,10]. Conversely, bioactive scaffolds such as hydrogels offer extracellular matrix–mimetic osteogenic cues but are mechanically weak and thus generally suitable for non-load-bearing defects [11,12]. Therefore, the development of advantageous scaffolds with mechanical support and biological functions accelerating tissue regeneration is crucially important for segmental bone repair [13,14].

Nature provides inspiration for addressing this challenge through hierarchically structured materials. Notably, porcupine quills exemplify a lightweight yet tough architecture that effectively resisting bending and damage [15]. Each quill consists of a rigid, high-density outer layer encasing a low-density porous foam with thin cell walls (~7.1 ± 1.1 μm) [16]. The quills exhibit high compressive buckling strength (167.9 ± 39.3 MPa) and elastic strain energy density (~14.3 MJ·m^−3^) prior to buckling [16,17]. The outer layer bears the majority of compressive loads, while the foam core absorbs energy and prevents catastrophic failure by accommodating the flexural deformation of the outer layer [18]. This hierarchical design with an outer dense wall and inner foam achieves an optimal balance of high mechanical strength and light weight, desirable for segmental bone scaffolds. Guided by these natural blueprints, a porcupine quill-inspired scaffold that couples a robust outer layer with an inner porous foam could simultaneously ensure mechanical integrity and offer internal porosity for tissue integration—an approach that remains rarely reported. Additive manufacturing enables the precise fabrication of biomimetic bone scaffolds with controllable porosity and has therefore been widely applied to the reconstruction of segmental bone defects [19,20]. However, most porous scaffolds made by additive manufacturing show regular, interconnected grid-like and gyroid patterns, lacking directional channels in the scaffold core for mass transportation [21,22]. This limits structural guidance for early vascular invasion and mass transport, thereby reducing repair efficiency for segmental bone defects. A crucial step to overcome this limitation is to incorporate internal medullary-like pathways that allow for the transportation of cells and fluids between the two sides of the segmental bone defect [23,24]. Thus, a dense outer layer of polyvinyl alcohol–glycerol (PG) can be fabricated by fused deposition modeling (FDM), while the inner chitosan (CS) foam with oriented pathways can be constructed via unidirectional freeze casting, thereby enabling the structural biomimicry of porcupine quills. Therefore, biomimeticCS/PG scaffolds inspired by porcupine quills likely provide both mechanical support and nutrient transportation for segmental bone defect repair.

Beyond architecture, however, the regenerative outcome of segmental bone injuries is frequently limited by insufficient vascularization and poor re-innervation. Zinc (Zn) is a trace element well-known to stimulate bone regeneration and angiogenesis. Zn^2+^-doped biomaterials have been shown to promote endothelial cell proliferation and extracellular matrix formation essential for new vessel growth [25,26,27]. Notably, amorphous zinc phosphate (AZP), a metastable nanocluster phase, can efficiently release Zn^2+^ ions, which has been shown to promote angiogenesis by upregulating the HIF-1α/VEGF pathway [28,29,30]. Additionally, poly (3,4-ethylenedioxythiophene) polystyrene sulfonic acid (PEDOT:PSS, denoted as PP) has attracted significant attention in neural tissue engineering owing to its excellent biocompatibility and ability to support the electrical stimulation of cells [31,32,33]. Conductive scaffolds create an electroactive microenvironment that can facilitate neurite extension and Schwann cell activity [34,35]. Even without external stimulation, the presence of a conductive PP can improve neural cell attachment and differentiation by mediating endogenous bioelectric signals [36,37]. Therefore, the incorporation of AZP and PP into the CS foams of the biomimetic CS/PG structure can further facilitate vascular and neural regeneration for segmental bone repair.

**Scheme 1:**
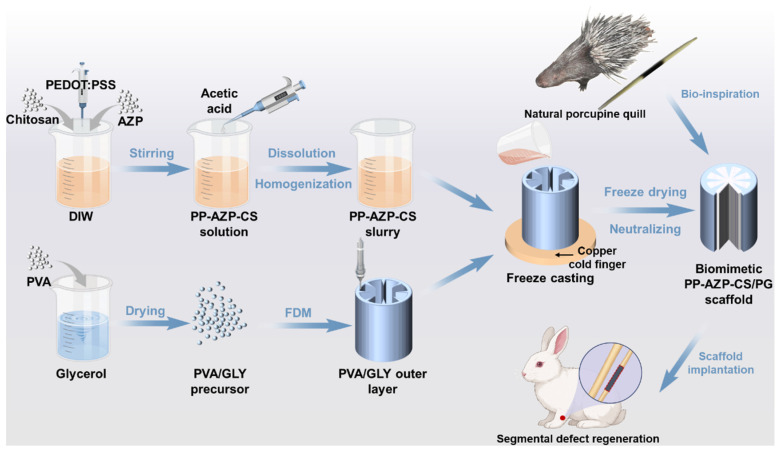
Schematic illustrations of fabrication of biomimetic PP-AZP-CS/PG scaffold inspired by *Hystrix* porcupine quill for segmental bone defect repair.

Herein, we constructed a novel biomimetic PP-AZP-CS/PG scaffold inspired by porcupine quills that aims to address major clinical challenges in segmental bone defect repair. Through FDM and unidirectional freeze casting strategies, the biomimetic hierarchical structure was developed featuring an outer load-bearing PG layer and an inner oriented porous CS foam with structure-guided functionality. By integrating PP and AZP within the CS foam, the scaffold acquired targeted conductive and angiogenic functions. In vitro assays were performed to evaluate the biocompatibility of the biomimetic scaffolds, as well as their ability to promote angiogenesis and osteogenic differentiation. In addition, a New Zealand white rabbit radial segmental defect model was established to assess the in vivo bone repair performance of the scaffolds. By constructing biomimetic PP-AZP-CS/PG scaffolds that combine porcupine quill-mimetic architecture and bioactive functionalization, we provide a promising strategy for segmental bone defect healing and regenerative medicine (Scheme 1).

## 2. Materials and Methods

### 2.1. Materials

Chitosan (CS, (C_6_H_11_NO_4_)_n_, deacetylation: 90–95%), polyvinyl alcohol (PVA, 99% hydrolyzed), and glycerol (AR, 99%) were purchased from Aladdin (Shanghai, China). Poly (3,4-ethylenedioxythiophene) polystyrene sulfonic acid (PEDOT:PSS) dispersion (1.3 wt% in water, conductive grade) was obtained from Sigma-Aldrich (St. Louis, MO, USA). Acetic anhydride (C_4_H_6_O_3_, AR, Mw = 102.09) and ethanol (C_2_H_5_OH, AR, Mw = 46.07) were provided by Sinopharm Chemical Reagent Co., Ltd. Polyacrylic acid (PAA, Mw ~ 2000 Da) and zinc chloride (ZnCl_2_, AR) were purchased from Bio-Chem Technology Co., Ltd. (Shanghai, China). Ammonia solution (NH_3_⋅H_2_O, 25–28 wt%), sodium hydroxide (NaOH, AR, ≥96.0 wt%), and diammonium hydrogen phosphate ((NH_4_)_2_HPO_4_, AR, ≥99.0 wt%) were purchased from Sinopharm Chemical Reagent Co. Ltd. (Shanghai, China). *Hystrix* porcupine quills were obtained from Xiangtai Porcupine Specialty Breeding Farm (Xiangxiang, China). All porcupine quills used were naturally shed by porcupines (body length 50–80 cm, age approximately 4–10 years).

### 2.2. Fabrication of Biomimetic PP-AZP-CS/PG Scaffold

Fabrication of PG outer layer: A total of 30 g of PVA was added to 20 g of GLY and continuously stirred for 30 min. The resulting mixture was then homogenized at 1800 rpm for 3 min under vacuum to ensure uniform mixing. Subsequently, the mixture was sealed and dried in an oven at 80 °C for 24 h, yielding PVA/GLY (PG) composite powder. An FDM printer (Regenovo, Hangzhou, China) was employed to fabricate the PG outer layer. Initially, the PG powder was loaded into the material chamber and fully melted at 240 °C. The molten PG composite was extruded through a 1.5 mm diameter nozzle at a rate of 0.5 mm/min to produce PG filaments. The preheated filaments were then fed back into the material chamber and fully remelted at 260 °C, and the melt was extruded through a 0.3 mm nozzle to construct a hollow PG outer layer with dimensions of φ14 mm × 7 mm. The fabricated PG hollow scaffolds were subjected to an etherification process in a vacuum oven. To optimize the mechanical strength of the scaffolds, a systematic screening of processing parameters was conducted, including three heating temperatures (120 °C, 140 °C, and 160 °C) and three time points (12, 24, and 48 h).

Synthesis of AZP nanoparticles: A total of 4.08 g of ZnCl_2_ and 1.28 g of PAA were dissolved in 500 mL of deionized water (DIW), and the pH was adjusted to 3.0 using NH_3_·H_2_O to obtain solution A. Separately, 2.64 g of (NH_4_)_2_HPO_4_ and 0.20 g of PAA were dissolved in 500 mL of DIW, and the pH was adjusted to 9.0 with NH_3_·H_2_O to obtain solution B. Solution A was then added dropwise to solution B under continuous stirring (500 rpm), and the mixture was stirred for an hour. The resulting dispersion was dialyzed against DIW (Mw cut-off 10 kDa) for 3 days. Finally, the AZP nanoparticles were collected by freeze-drying (Gold SIM International Group, Miami, FL, USA).

Preparation of PP-AZP-CS slurry: A total of 1.75 g of CS powder, 0.0175 g of AZP powder, and 6.79 mL of PP dispersion were mixed with 42.27 mL of DIW, and 1mL of glacial acetic acid was rapidly dripped under 1500 rpm stirring conditions at room temperature for 20 min and placed in a vacuum mixer for defoaming, resulting in a slurry containing 1% (*w*/*w*) AZP, 5% (*w*/*w*) PP, and 3.5% (*w*/*v*) CS. The 2% and 5% (*w*/*v*) CS slurries were prepared by adding different amounts of AZP, PP and CS powder in the same procedure. The slurry was stored at 4 °C for subsequent use.

Assembly of biomimetic PP-AZP-CS/PG scaffold: A biomimetic PP-AZP-CS/PG scaffold was fabricated by the unidirectional freeze casting of the slurry infused into the PG outer layer. Unidirectional freeze casting was conducted using liquid nitrogen as the temperature source and a copper cold finger as the thermal conductor. The temperature was maintained at −80 °C for 30 min via a PID controller (Yudian Automation Technology Co., Ltd., Xiamen, China) until complete solidification. The scaffolds were then transferred to a −20 °C freezer for 24 h to complete phase separation, followed by lyophilization at −60 °C for 48 h to obtain PP-AZP-CS/PG scaffolds. To neutralize the residual acetate byproducts formed during synthesis, the scaffolds were immersed in 0.1 M NaOH solution for 15 min, followed by rinsing with DIW three times to eliminate excess NaOH.

### 2.3. The Physicochemical Characterization of the Biomimetic PP-AZP-CS/PG Scaffolds

#### 2.3.1. Phase Structure and Crystallinity

The crystallization characteristics of the amorphous nanoparticles and composites were assessed using an X-ray diffractometer (XRD, SmartLab, Tokyo, Japan) equipped with Cu Kα radiation (λ = 1.5406 Å), operating at 40 kV and 100 mA, across a 2θ range of 20° to 70°. The morphology and crystallinity were analyzed using transmission electron microscopy (TEM, JEM-2100plus, JEOL Ltd., Tokyo, Japan) and selected area electron diffraction (SAED) with TEM.

#### 2.3.2. Microstructure and Elemental Distribution

The surface morphology and elemental composition of the scaffolds were analyzed using a scanning electron microscope (SEM, Merlin Compact, Zeiss, Jena, Germany) coupled with energy-dispersive spectroscopy (EDS, Oxford Instruments, Abingdon, UK). The scaffolds were prepared for SEM examination by sputtering and gold-plating using a sputtering system (JFC-1200, JEOL, Tokyo, Japan).

#### 2.3.3. Chemical Composition and Bonding

To further investigate the material composition, Fourier transform infrared spectroscopy (FT-IR, Cary 630, Agilent Technologies, Santa Clara, CA, USA) was used, with a spectral range from 4000 to 400 cm^−1^ and a resolution of 4 cm^−1^.

#### 2.3.4. Thermal Stability and Composition

Thermal properties were measured by a thermogravimetric analyzer (TGA, Netzsch STA 449F3, Netzsch, Selb, Germany), and the samples were heated from 50 °C to 1000 °C at a rate of 5 °C/min.

#### 2.3.5. Hydration Behavior and Surface Wettability

The swelling behavior of the scaffolds was determined by a gravimetric method. Briefly, samples were dried at 50 °C to constant mass and weighed to obtain the initial dry weight (*W_dry_*). The dried samples were then immersed in PBS. At predetermined time points (0.5, 1, 2, 4, 8, 16, and 32 h), the samples were removed, gently blotted with filter paper to eliminate surface water, and immediately weighed to record the wet weight (*W_i_*). The swelling ratio (*SR*) of the PG composite material was calculated as follows:

$$SR=\frac{W_{i}-W_{dry}}{W_{dry}}$$

Surface hydrophilicity was determined using the OCA20_U contact angle system (DadaPhysics Instruments GmbH, Stuttgart, Germany). The volume of DIW droplets used for the measurement was 3 μL, and the data were recorded within 10 s of dropping. The average value was obtained by three measurements taken at different positions.

#### 2.3.6. Degradation Behavior

The degradation behavior of the scaffolds was evaluated in PBS (pH = 7.4) containing lysozyme (10,000 U/mL). All samples were dried at 50 °C to constant mass and weighed to obtain the initial dry weight (*W_dry_*). Subsequently, each sample was immersed in 10 mL of the degradation medium in a test tube and incubated in a shaking incubator at 37 °C. The medium was refreshed daily to maintain a consistent degradation environment. Samples were collected at predetermined time points (1, 3, 7, and 14 days). At each time point, the samples were removed, rinsed thoroughly with deionized water, and freeze-dried for 8 h. The dry weight at each time point was recorded as *W_t_*. The in vitro degradation rate (*DR*) was calculated as follows:

$$DR=\left(W_{dry}-W_{t}\right)∕W_{dry}$$

#### 2.3.7. Porosity and Pore Interconnectivity

The porosity of the foams was determined using an ethanol displacement method. First, the foams were weighed to obtain the dry weight (*W_dry_*). The dimensions of the foams were measured using a vernier caliper to calculate their volume (*V_foam_*). The foams were then placed in a beaker filled with an excess of anhydrous ethanol and subjected to vacuum treatment in a drying oven for 5 min per sample until fully submerged. Residual ethanol was gently blotted from the foam surface with filter paper, and then *W_1_* was recorded. Porosity (*P*) was calculated using the following formula:

$$P=\frac{V_{pore(ethanol)}}{V_{foam}}=\frac{W_{1}-W_{dry}}{\rho_{ethanol}V_{foam}}$$

### 2.4. Measurement of Mechanical Properties of Biomimetic PP-AZP-CS/PG Scaffolds

Different etherification times and temperatures significantly influence the mechanical properties of composite materials. The compressive mechanical properties of these materials were assessed using a universal testing machine (ZQ 990 LB, Zhiqu Precision Instruments Co., Dongguan, China). The samples were tested at a compression rate of 0.5 mm/min. Stress–strain curves were used to calculate the elastic modulus (strain 0–25%) and yield strength.

### 2.5. Cell Culture

Mouse embryo osteoblast precursor cells (MC3T3-E1) were purchased from the Cell Bank of the Chinese Academy of Sciences (Shanghai, China). MC3T3-E1 cells were cultured in the α-MEM medium (Hyclone, GE Healthcare, Chicago, IL, USA) supplemented with 10% FBS (Lonsera, Ciudad de la Costa, Uruguay) and 1% penicillin–streptomycin (PS, Genview Scientific, Inc., Calimesa, CA, USA). Human umbilical vein endothelial cells (HUVECs) were obtained from the American Type Culture Collection (ATCC, Manassas, VA, USA) and cultured in high-glucose Dulbecco’s Modified Eagle Medium (H-DMEM) supplemented with 10% FBS and 1% PS. All cells were cultured in their respective media, which were refreshed every 2 days, under standard conditions of 37 °C and 5% CO_2_.

### 2.6. Cell Viability and Proliferation

Scaffold extracts were prepared by incubating sterilized samples in complete culture medium (10% FBS and 1% PS) at a ratio of 0.05 g/mL. Extraction was conducted at 37 °C with orbital shaking at 80 rpm for 72 h. The supernatants were then collected and sterilized by filtration through a 0.22 μm membrane (aqueous, PES) to obtain the extracts used for subsequent cell assays.

CCK-8 Assay: After cell seeding, cells were cultured at 1, 3, and 5 days in extracts of different scaffolds. A 10% Cell Counting Kit-8 reagent (CCK-8, Genview Scientific, Inc., Calimesa, CA, USA) was added, and after incubation for 2 h, cell viability was measured using a microplate reader (Multiskan Go, Thermo Fisher Scientific, Waltham, MA, USA).

Live/Dead Staining: Cells cultured in extracts of different scaffolds for 1 and 3 days were incubated with the live/dead cell staining solution for 15 min and observed under a fluorescence microscope (Nikon Instruments Inc., Tokyo, Japan). Green fluorescence indicated living cells, while red fluorescence indicated dead cells.

### 2.7. Effect of Scaffolds on Angiogenesis In Vitro

Migration assay using scratch wound model: HUVECs were seeded at a density of 2 × 10^5^ cells per well in a 24-well plate and cultured with serum-free DMEM. The medium in each well was replaced with 1 mL of the extracts of different scaffolds after the cells were confluent in the well plates under the microscope. A vertical scratch was created in each well using a 200 μL pipette tip, and the starting line was designated as the initial time point. Cell migration was photographed at 0, 6, and 12 h under a microscope. The captured images were analyzed quantitatively using ImageJ 1.54p software.

Tube formation assay: Matrigel (50 μL, Corning, Corning, NY, USA) was injected into pre-chilled 24-well plates. HUVECs were resuspended in extracts from different scaffolds, with 2 × 10^5^ cells seeded per well. Newly formed tubular structures were photographed under a microscope at 0, 6 and 12 h. Tubular structures were quantified using ImageJ software.

### 2.8. Effect of Scaffolds on Osteogenic Differentiation

Alkaline phosphatase (ALP) staining: MC3T3-E1 cells were cultured for 7 and 10 days with extracts from different scaffolds prepared using osteogenic induction medium (consisting of 10 mM β-glycerophosphate, 50 µg/mL ascorbic acid, and 10 nM dexamethasone in α-MEM supplemented with 10% FBS). Cells were fixed with 4% paraformaldehyde for 30 min and washed three times with PBS. The samples were then stained using an ALP assay kit (Beyotime, Shanghai, China) for an hour and imaged under a fluorescence microscope. The semi-quantitative evaluation of ALP activity was performed according to the instructions of the ALP detection kit (E1040, Applygen, Beijing, China).

Alizarin red S (ARS) staining: MC3T3-E1 cells were cultured for 7 and 10 days with extracts from different scaffolds prepared using osteogenic induction medium (consisting of 10 mM β-glycerophosphate, 50 µg/mL ascorbic acid, and 10 nM dexamethasone in α-MEM supplemented with 10% FBS). Cells were fixed with 4% paraformaldehyde solution for 30 min. After rinsing with PBS three times, the cells were stained with an ARS kit (Beijing Solarbio Science and Technology Co., Ltd., Beijing, China) for 10 min, thoroughly rinsed with DIW, and imaged under a fluorescence microscope. For quantitative analysis, 10% hexadecyl pyridinium chloride monohydrate (Sigma-Aldrich, St. Louis, MO, USA) was used to dissolve the mineralized nodules, and the OD value was measured at 562 nm.

### 2.9. The Implantation of the Scaffolds in Bone Defects

Implantation for segmental defects of the radial bone in New Zealand white rabbits: All animal procedures were approved by the Experimental Animal Ethics Committee of Jinzhou Medical University (Approval No. 230211-5) and conducted in accordance with the institution’s guidelines for the care and use of laboratory animals. Experimental animals were healthy New Zealand white rabbits (6–8 months old, weighing 2–3 kg, with equal numbers of males and females), purchased from the Experimental Animal Center of Jinzhou Medical University. Animals were randomly divided into three groups: (1) blank; (2) CS/PG scaffold; and (3) PP-AZP-CS/PG scaffold. After one week of acclimatization, surgery was performed under general anesthesia induced by the intravenous injection of tiletamine hydrochloride (10 mg·kg^−1^) and zolazepam hydrochloride (10 mg·kg^−1^). Under sterile conditions, a 6 mm segmental defect was prepared in the mid-radius, and the periosteum over the defect site was carefully stripped. The corresponding scaffolds were implanted into the defect site to completely fill the space. The postoperative condition of each animal was monitored regularly. The rabbits were sacrificed at 4 and 8 weeks post-surgery, and the harvested radial specimens were fixed in 4% paraformaldehyde for subsequent micro-CT and histological analyses. At both 4 and 8 weeks, the sample size was *n* = 4 per group.

Micro-CT analysis: Representative images were obtained by micro-CT (Skyscan 1276, Bruker micro-CT, Kontich, Belgium). The scanning parameters were an effective pixel size of 20.24 μm, a current of 200 μA, a voltage of 70 kV, and an exposure time of 388 ms. The 2D images were reconstructed into 3D images by Bruker CTAn analysis software to calculate the bone regeneration parameters: bone volume/total volume (BV/TV), trabecular thickness (Tb.Th), and trabecular number (Tb.N).

### 2.10. Statistical Analysis

All quantitative data are presented as the mean ± standard deviation (SD), with each experiment performed at least in triplicate. Comparisons among groups for single-time-point measurements were conducted using one-way analysis of variance (ANOVA) followed by Tukey’s post hoc test, whereas data involving multiple time points were analyzed using two-way ANOVA with Tukey’s post hoc test. Statistical significance was set at *p* < 0.05.

## 3. Results and Discussion

### 3.1. Fabrication of Biomimetic PP-AZP-CS/PG Scaffold Inspired by Natural Hystrix Porcupine Quills

**Figure 1:**
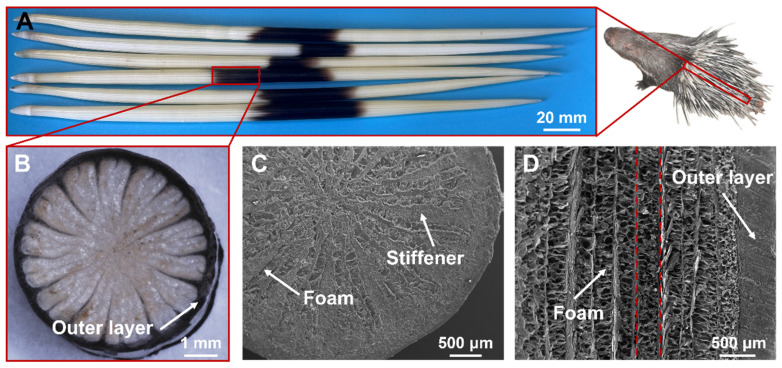
Characterization of natural *Hystrix* porcupine quills. (**A**) Photo of *Hystrix* porcupine quills. (**B**) Cross-sectional photo of *Hystrix* porcupine quill. (**C**) SEM image of cross-section of *Hystrix* porcupine quill. (**D**) SEM image of longitudinal cross-section of *Hystrix* porcupine quill, the sections between the red dashed lines represent a directional channel.

Natural *Hystrix* porcupine quills are keratin-based biomaterials that serve as key defensive organs against predators due to their lightweight and high strength characteristics, which originate from their complex hierarchical structure (Figure 1A and Figure S1) [16,17,18]. As observed from the transverse cross-section of the natural quill (Figure 1B), the cylindrical outer layer comprises a dense shell, whereas the inner layer exhibits a loose and porous foam morphology (Figure 1C). In particular, the stiffener structure extends from the outer shell into the inner core, representing a typical structural feature of *Hystrix* porcupine quill [16]. Moreover, SEM images of the longitudinal section revealed that the inner foam contained oriented channels with a characteristic size of approximately 260 μm (Figure 1D), which effectively prevented brittle fracture after load application [17]. Notably, the structure and function of natural porcupine quills resembled those of bone tissues. The dense outer shell provided mechanical support and protection, analogous to the role of cortical bone, while the oriented inner foam formed a loose, porous architecture like cancellous bone [38].

**Figure 2:**
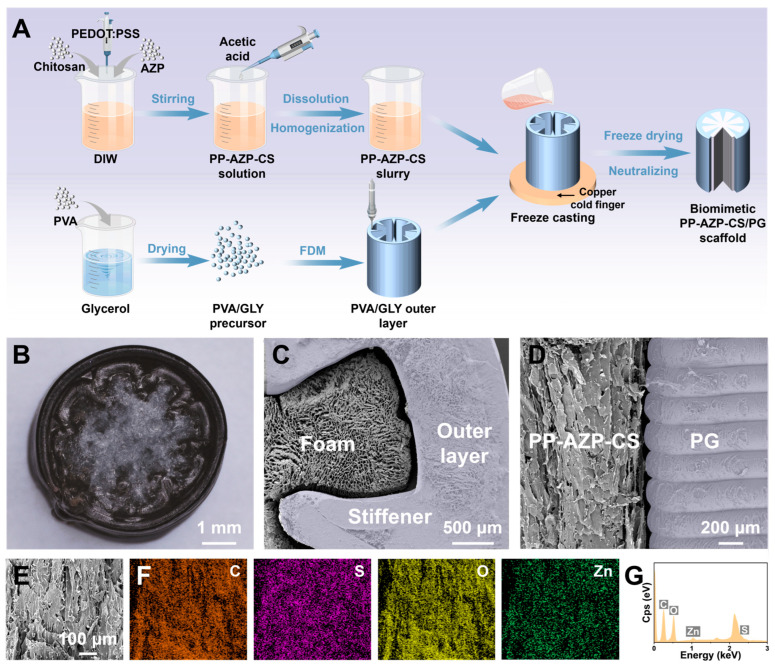
Characterization of biomimetic PP-AZP-CS/PG scaffold. (**A**) Schematic illustration for fabrication process of biomimetic scaffold inspired by *Hystrix* quills. (**B**) Photo of cross-sectional scaffold. (**C**) SEM image of cross-section of scaffold (outer layer is highlighted in artificial purple color). (**D**) SEM image of longitudinal cross-section of scaffold (outer layer is highlighted in artificial purple color). (**E**) SEM image of longitudinal cross-section of inner PP-AZP-CS foam. (**F**) Elemental distribution of longitudinal cross-section of inner PP-AZP-CS foam. (**G**) EDS spectrum of longitudinal cross-section of inner PP-AZP-CS foam.

Building on the hierarchical structural advantages of natural *Hystrix* porcupine quills, this study combined FDM with unidirectional freeze casting to construct a biomimetic PP-AZP-CS/PG scaffold, as illustrated in Figure 2A. The photo (Figure 2B) and SEM image (Figure 2C) of the scaffold cross-section showed that the as-prepared scaffold exhibited distinct hierarchical structural features. The outer layer consisted of a dense stiffener–shell structure, while the inside was a loose, porous foam. The interface between the two layers was tightly bonded with a smooth transition. The longitudinal cross-section revealed vertically aligned, directed channels in the inner layer (Figure 2D). The elemental mapping results indicated that C, O, S, and Zn elements were uniformly distributed within the inner PP-AZP-CS foam, confirming the effective incorporation of active components (Figure 2E–G). The overall structure of the biomimetic PP-AZP-CS/PG scaffold closely matched the hierarchical features of the natural *Hystrix* porcupine quill.

**Figure 3:**
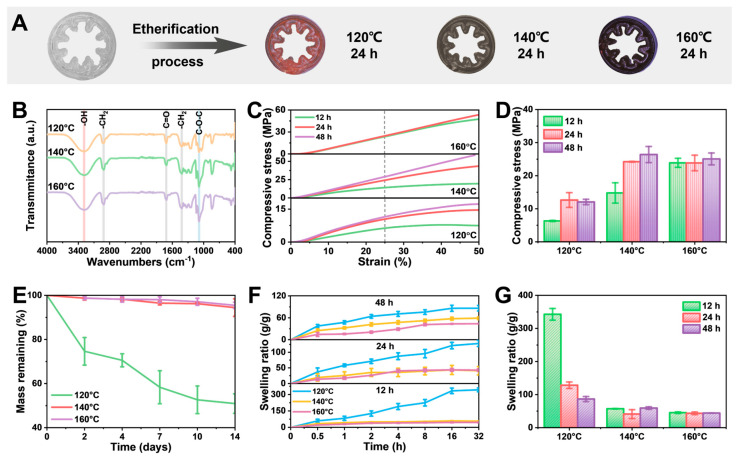
Etherification process of PG composites. (**A**) Macroscopic morphology of PG-120 °C/24 h, PG-140 °C/24 h, and PG-160 °C/24 h. (**B**) FT-IR spectra of PG-120 °C/24 h, PG-140 °C/24 h, and PG-160 °C/24 h. (**C**) Uniaxial compressive stress–strain curves of PG composites with different etherification parameters. (**D**) Compressive strength of PG composites with different etherification parameters at 25% strain. (**E**) In vitro degradation curves of PG-120 °C/24 h, PG-140 °C/24 h, and PG-160 °C/24 h in lysozyme–PBS solution. **(F**) In vitro swelling curves of PG composites with different etherification parameters in PBS. (**G**) Swelling ratios of PG composites with different etherification parameters after 32 h.

The fabrication details of the biomimetic scaffold are as follows: PG composites were prepared via FDM to construct the biomimetic outer layer structure of the scaffold, with the printing parameters listed in Table S1. To enhance the structural stability and mechanical support of the layer, the printed PG composites were subsequently subjected to etherification at different temperatures. After the process, the as-prepared PG layer retained robust macroscopic structural integrity, but noticeable color changes were observed. The color darkened gradually with increasing etherification temperature, from a brownish yellow at 120 °C to a dark brown at 160 °C (Figure 3A). Additionally, FT-IR analysis was used to investigate the chemical bonding of etherification on the PG composite (Figure 3B). The results showed that PG composites exhibited absorption peaks at 3276 cm^−1^, 2918 cm^−1^, 1423 cm^−1^, 1716 cm^−1^, and 1042 cm^−1^ at all temperatures, corresponding to hydroxyl stretching vibration, –CH_2_ asymmetric stretching, –CH_2_ symmetric bending, C=O stretching vibration, and C–O–C stretching vibration, which are characteristic of PVA and GLY peaks. Notably, as the etherification temperature increased, the intensity of the C–O–C bond absorption peak at 1042 cm^−1^ increased, while the O–H stretching vibration peak at 3276 cm^−1^ significantly weakened. This trend indicated that a larger fraction of hydroxyl groups underwent dehydration to form ether linkages, resulting in an enhanced degree of etherification, consistent with reports for other PVA-based systems [39]. Additionally, the influence of PG etherification degree on mechanical properties was further analyzed by conducting uniaxial compression tests on PG samples prepared under different conditions. Figure 3C,D show the stress–strain curves and the compressive strength at 25% strain, respectively. The results indicated that the compressive strength of PG composites generally increased with extended processing time at 120 °C, though minimal differences were observed at 24 h (12.61 ± 2.23 MPa) and 48 h (12.03 ± 0.81 MPa). When the temperature was elevated to 140 °C, compressive strength further increased, reaching 24.21 ± 0.11 MPa and 26.38 ± 2.44 MPa at 24 and 48 h, respectively. In contrast, no significant change in strength was observed for PG composites processed at 160 °C. Detailed values for the compressive strength of various samples are provided in Table S2. These results suggested that the increase in the compressive strength of PG after etherification was mainly due to the formation of ether bonds through hydroxyl dehydration, which led to structural compaction. Meanwhile, the etherification process significantly improved the structural stability of PG composites. To evaluate in vitro degradation behavior, accelerated degradation experiments were conducted using PBS containing lysozyme. As shown in Figure 3E, under the same process time (24 h), increasing the temperature significantly reduced the in vitro degradation rate of the samples. When the temperature was raised from 120 °C to 140 °C, the remaining mass of the samples increased from 50.93 ± 4.41% to 94.45 ± 3.97% after immersion for 14 days. However, no significant change occurred when the temperature further increased to 160 °C. Furthermore, the swelling behavior of PG in PBS is shown in Figure 3F. When the temperature exceeded 140 °C, the swelling rate of the samples stabilized in the range of 40–50% after 32 h, demonstrating excellent anti-swelling performance. Therefore, the etherification of PG effectively decreased the hydrophilicity of PG, resulting in significantly low swelling [40].

**Figure 4:**
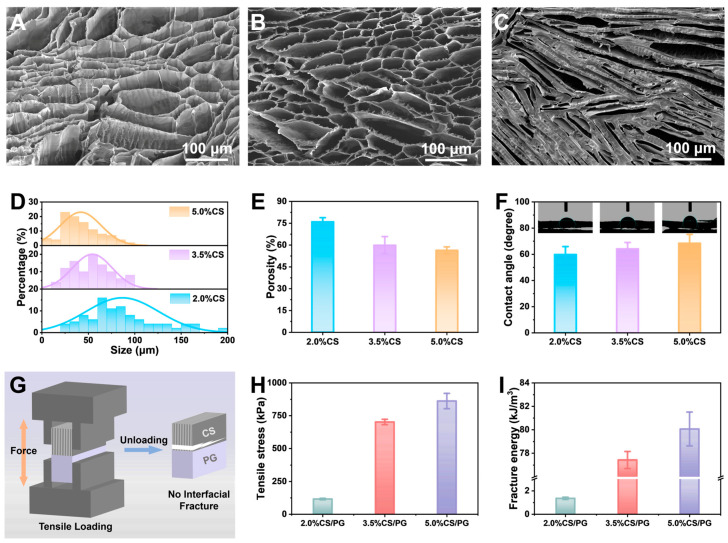
Characterization of CS foams at different concentrations. (**A**) SEM image of cross-section of 2% CS foam. (**B**) SEM image of cross-section of 3.5% CS foam. (**C**) SEM image of cross-section of 5% CS foam. (**D**) Pore size distribution of 2%, 3.5%, and 5% CS foams. (**E**) Porosity of CS foams at 2%, 3.5%, and 5% concentrations. (**F**) Surface hydrophilicity of CS foams at 2%, 3.5%, and 5% concentrations. (**G**) Schematic of CS/PG interface bonding strength testing. (**H**) Maximum tensile strength of 2% CS/PG, 3.5% CS/PG, and 5% CS/PG. (**I**) Fracture energy of 2% CS/PG, 3.5% CS/PG, and 5% CS/PG.

A biomimetic PP-AZP-CS foam structure inspired by the inner layer of *Hystrix* porcupine quills was fabricated via unidirectional freeze casting technology. This technology has been proven effective in guiding the alignment of fibers in a specific direction by controlling the cooling rate, temperature, direction and concentration of the slurry, resulting in a porous foam with unique structural characteristics [41]. Three different concentrations (2%, 3.5%, and 5%) of the CS slurry were used for freeze casting at −80 °C. The cooling source was placed below the CS slurry, and the unidirectional temperature gradient promoted the orderly arrangement of ice crystals along the axial direction, which in turn influenced the alignment of the CS fibers. SEM images of the cross-sections of the CS foams at different concentrations were obtained (Figure 4A–C). The results showed that all samples exhibited interconnected porous structures. However, as the concentration increased, the pore structure became more compact. Specifically, 2.0% CS exhibited larger, more open pores with thinner pore walls; 3.5% CS displayed a reduction in both pore size and connectivity; and 5.0% CS showed thicker pore walls, smaller pores, and a denser structure. Quantitative analysis further demonstrated that the pore size distribution shifted to smaller values with increasing CS concentration (Figure 4D). The 2.0% CS sample exhibited a broader distribution (80–100 µm), whereas the 3.5% and 5.0% CS samples showed a left-shifted and more concentrated distribution (50–60 µm and 35–45 µm), indicating reduced pore size and improved uniformity. Pore size quantification showed a gradual decrease in porosity as CS concentration increased, from 76.0% in the 2.0% CS group to 56.4% in the 5.0% CS group (Figure 4E). High CS concentrations increased the viscosity and solid content of the slurry, enhancing fiber and pore wall aggregation during freeze casting, thus forming a more compact pore structure. In addition, the effective degradation of the inner foam allowed for the sustained release of the active AZP and PP components. In vitro degradation tests revealed a rapid mass loss during the initial 2 days, followed by a gradual decline rate. After 14 days, the remaining mass percentages for each group were 81.13 ± 1.14%, 81.17 ± 1.34%, and 81.20 ± 1.18%, indicating that varying CS concentration did not significantly affect the water-mediated stability of the foam structure (Figure S2). A quantitative analysis of the hydrophilic properties of the foams was undertaken by measuring the static contact angle of water drops on the surface. The measured contact angles for 2.0% CS, 3.5% CS, and 5.0% CS were approximately 59.77°, 64.17°, and 68.43°, respectively. An increase in CS content resulted in an elevated contact angle, while the hydrophilic nature of the samples was retained (Figure 4F). Furthermore, the capillary wicking behavior of the foam was visualized. The red hydrophilic ink rapidly rose to the top of the foam within 3 s, indicating efficient liquid uptake through the oriented channels (Figure S3). The interface binding strength was quantitatively evaluated under tensile loading (as shown in Figure 4G), in order to investigate the effect of CS concentration on the interfacial bonding between the outer layer and foam. The 5.0% CS/PG samples exhibited markedly larger fracture displacement during the tensile test compared to the other two groups (Figure S4). The maximum tensile strength of 5.0% CS/PG samples reached 859.33 ± 157.42 kPa, significantly higher than that of 2.0% CS/PG (115.32 ± 5.69 kPa) and 3.5% CS/PG (272.53 ± 96.88 kPa) (Figure 4H). Similarly, the fracture energy of the 5% CS/PG sample was 79.40 ± 2.09 kJ/m^3^, substantially exceeding that of the 2.0% CS/PG and 3.5% CS/PG samples (Figure 4I). Overall, an inner CS foam concentration of 3.5% achieved a favorable balance by maintaining the biomimetic porous structure while providing the optimal interfacial bonding strength.

A *Hystrix* porcupine quill-mimetic architecture was constructed by integrating FDM with unidirectional freeze casting. The etherification of the PG composites was used to adjust the physicochemical properties and mechanical performance of the biomimetic outer layer to meet the requirements of segmental bone defect repair. Notably, the hydrophilic, oriented channel foam not only provided structural support but also markedly enhanced rapid wetting and capillary wicking, which may facilitate nerve ingrowth and blood perfusion along the aligned channels from both ends of the defect. Moreover, the oriented architecture promotes cell adhesion and directional growth, enabling subsequent biomodification to further support specific functional expression [42,43]. Consequently, the biomimetic porcupine quill structure provided mechanical support while creating a more favorable segmental bone regeneration environment by establishing hydrophilic, oriented channels.

**Figure 5:**
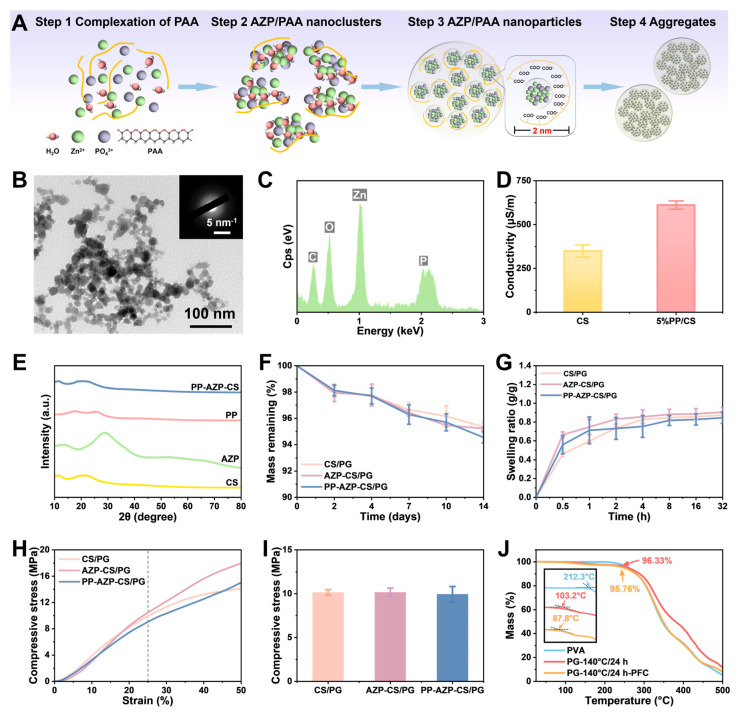
Characterization of biomimetic AZP–PP–CS/PG scaffold. (**A**) Schematic of AZP synthesis. (**B**) TEM and electron diffraction patterns of AZP. (**C**) XRD pattern of AZP sample. (**D**) Conductivity of CS and 5% PP-CS samples. (**E**) XRD patterns of PP, AZP, CS, and PP-AZP-CS samples. (**F**) In vitro degradation curves of CS/PG, AZP-CS/PG, and PP-AZP-CS/PG. (**G**) In vitro swelling curves of CS/PG, AZP-CS/PG, and PP-AZP-CS/PG. (**H**) Axial compressive stress–strain curves of CS/PG, AZP-CS/PG, and PP-AZP-CS/PG, the gray dashed line represents the compressive stress at 25% strain. (**I**) Compressive strength of CS/PG, AZP-CS/PG, and PP-AZP-CS/PG at 25% strain. (**J**) TGA patterns of PVA and PG-140 °C/24h (before and after freeze casting) from 20 °C to 500 °C.

During the preparation of the CS slurry, bioactive components (AZP and PP) were incorporated to enhance the pro-angiogenic and pro-neurogenic potential of the biomimetic scaffold. Figure 5A illustrates the synthesis process of AZP nanoparticles. Briefly, the Zn^2+^ solution, thoroughly mixed with PAA, was slowly added to a phosphate solution. Zn^2 +^ complexed with PAA molecules, while the electrostatic interaction between PO_4_^3−^ and Zn^2+^ facilitated the formation of initial AZP/PAA nanoclusters. These nanoclusters underwent PAA-mediated self-assembly and gradually aggregation, ultimately forming AZP aggregates [28,29,30]. TEM analysis showed that AZP existed as individual nanoparticles with a characteristic size of ~10 nm, together with small aggregates (Figure 5B). The corresponding SAED pattern displayed diffuse rings with indistinct boundaries, confirming the amorphous state of AZP. EDS analysis further indicated a Zn:P atomic ratio of 1.62 in the AZP nanoparticles, consistent with our previous findings (Figure 5C) [28,29,30]. Furthermore, 5 wt% PP was incorporated into the CS foam (5%PP/CS). The electrical conductivity measurements conducted using a digital multimeter demonstrated a significant increase in conductivity from 349.48 ± 35.09 μS/m to 611.54 ± 22.55 μS/m (Figure 5D and Figure S5), thereby indicating that PP incorporation effectively enhanced charge transport, crucially important for promoting neural growth. The phase composition of the CS, PP, AZP, and PP-AZP-CS samples was characterized by XRD (Figure 5E), indicating amorphous characteristics, which may be beneficial for subsequent ion release, in vivo degradation, and osteogenesis-related processes [44].

The physical and mechanical properties of the biomimetic scaffolds (CS/PG, AZP-CS/PG, and PP-AZP-CS/PG) were further evaluated. In vitro degradation behavior indicated that the degradation rates remained essentially consistent across all groups (Figure 5F). The mass retention rates for the CS/PG, AZP-CS/PG, and PP-AZP-CS/PG scaffold groups were 95.35%, 95.23%, and 94.55% after soaking for 14 days, respectively. Similarly, no significant differences were observed in the anti-swelling properties among the groups (Figure 5G). In compression testing, the compressive strength at 25% strain was comparable among the three scaffolds, approximately 10.15 ± 0.31 MPa (CS/PG), 10.17 ± 0.48 MPa (AZP-CS/PG), and 9.95 ± 0.87 MPa (PP-AZP-CS/PG). These values were slightly lower than that for the pure PG shell treated at 140 °C for 24 h (Figure 5H,I). TGA measurements were performed to assess the water/GLY mass loss and thermal stability of pure PVA and PG outer layers before and after freeze casting (Figure 5J). The experiment was conducted in air with a heating rate of 10 °C/min. The inset shows the onset mass loss results for the samples (the curves in the inset were arbitrarily shifted along the y-axis for better observation). Following GLY incorporation and freeze casting, the onset temperature of mass loss was progressively decreased in the samples, dropping from 212.3 °C for pure PVA to 103.2 °C for etherified PG-140 °C/24 h. For PG-140 °C/24 h-PFC (CS removal followed by post-freeze casting), the onset temperature of mass loss was further reduced to 87.3 °C. Upon heating, water and GLY were released first, and the degradation of the PVA backbone became evident at approximately 250 °C. During the initial stage of water and GLY removal (40–240 °C), PG-140 °C/24 h exhibited a mass loss of 3.67%, whereas PG-140 °C/24 h-PFC showed a higher weight loss of 4.24%. Collectively, these results indicated that freeze casting increased the retained water and glycerol content in PG, which likely contributed to the relatively low compressive strength of the PG scaffold.

Taken together, the biomimetic AZP-PP-CS/PG scaffold fabricated by FDM and unidirectional freeze casting exhibits an obvious *Hystrix* quill-mimetic hierarchical shell–stiffener–foam architecture. This scaffold maintains structural stability and mechanical support while enabling the release of bioactive components through controlled degradation.

### 3.2. In Vitro Assessment of Biological Functions

**Figure 6:**
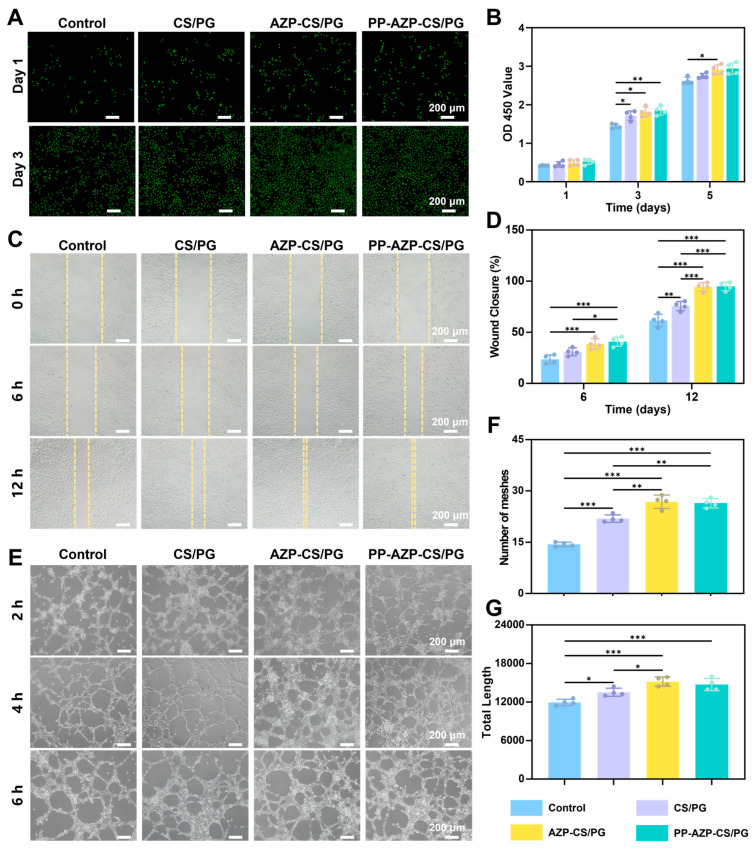
Effects of biomimetic PP-AZP-CS/PG scaffolds on HUVEC proliferation, migration, and angiogenesis. (**A**) Live/dead staining of HUVECs at 1 day and 3 days post-culture. (**B**) CCK-8 assay of HUVECs at 1, 3, and 5 days. (**C**) Representative scratch wound healing images of HUVECs at 0, 6, and 12 h, the yellow dashed line represents the boundary of the scratch. (**D**) Quantitative analysis of scratch wound healing at 0, 6, and 12 h. (**E**) Representative images of tube formation assay of HUVECs at 2, 4, and 6 h. (**F**) Quantitative analysis of number of meshes of blood vessels in tube formation assay of HUVECs at 2, 4, and 6 h. (**G**) Quantitative analysis of total length of blood vessels in tube formation assay of HUVECs at 2, 4, and 6 h. (* *p* < 0.05, ** *p* < 0.01, *** *p* < 0.001).

Vascular regeneration played a crucial role in the repair process of segmental bone defects by ensuring the oxygen and nutrient supply to the defect site and mediating osteogenesis-related signaling pathways, thereby providing essential microenvironmental support for new bone formation [45]. To evaluate the biocompatibility and pro-angiogenic potential of the biomimetic PP-AZP-CS/PG scaffolds, HUVECs were co-cultured with the scaffold extracts. Cell activity and proliferation were assessed via live/dead staining and CCK-8 assays (Figure 6A,B). No significant cytotoxicity was observed across all scaffold groups, with sustained cell proliferation and minimal dead cells. Specifically, compared to the CS group, the AZP-CS/PG and PP-AZP-CS/PG groups exhibited significantly higher cell proliferation on day 3 and day 5, indicating that the incorporation of AZP positively promoted the proliferation of HUVECs in the scaffold. A scratch wound assay was further performed to evaluate the effect of the scaffolds on HUVEC migration (Figure 6C). AZP-CS/PG and PP-AZP-CS/PG significantly enhanced HUVEC migration compared to the control and CS/PG, with the scratch area almost completely closed at 12 h, indicating improved vascular migration capabilities. Quantitative analysis showed that the wound closure rates of the AZP-CS/PG and PP-AZP-CS/PG groups reached approximately 95% at 12 h, markedly exceeding those of the control (61.59%) and CS/PG (75.66%) groups (Figure 6D). In addition, the in vitro tube formation assay revealed that the AZP-CS/PG and PP-AZP-CS/PG groups formed denser and more continuous tubular networks at all time points, indicating that the Zn-containing components significantly promoted the endothelial vascularization phenotype (Figure 6E). Consistently, quantitative analysis demonstrated that AZP-CS/PG and PP-AZP-CS/PG exhibited significantly higher numbers of meshes per mm^2^ and greater tube length than the CS/PG and control groups at 6 h (Figure 6F,G). These results confirmed that the AZP-containing scaffolds (AZP-CS/PG and PP-AZP-CS/PG) effectively enhanced angiogenesis, mainly due to the Zn^2+^ released from AZP, consistent with our previous work [28,29,30].

**Figure 7:**
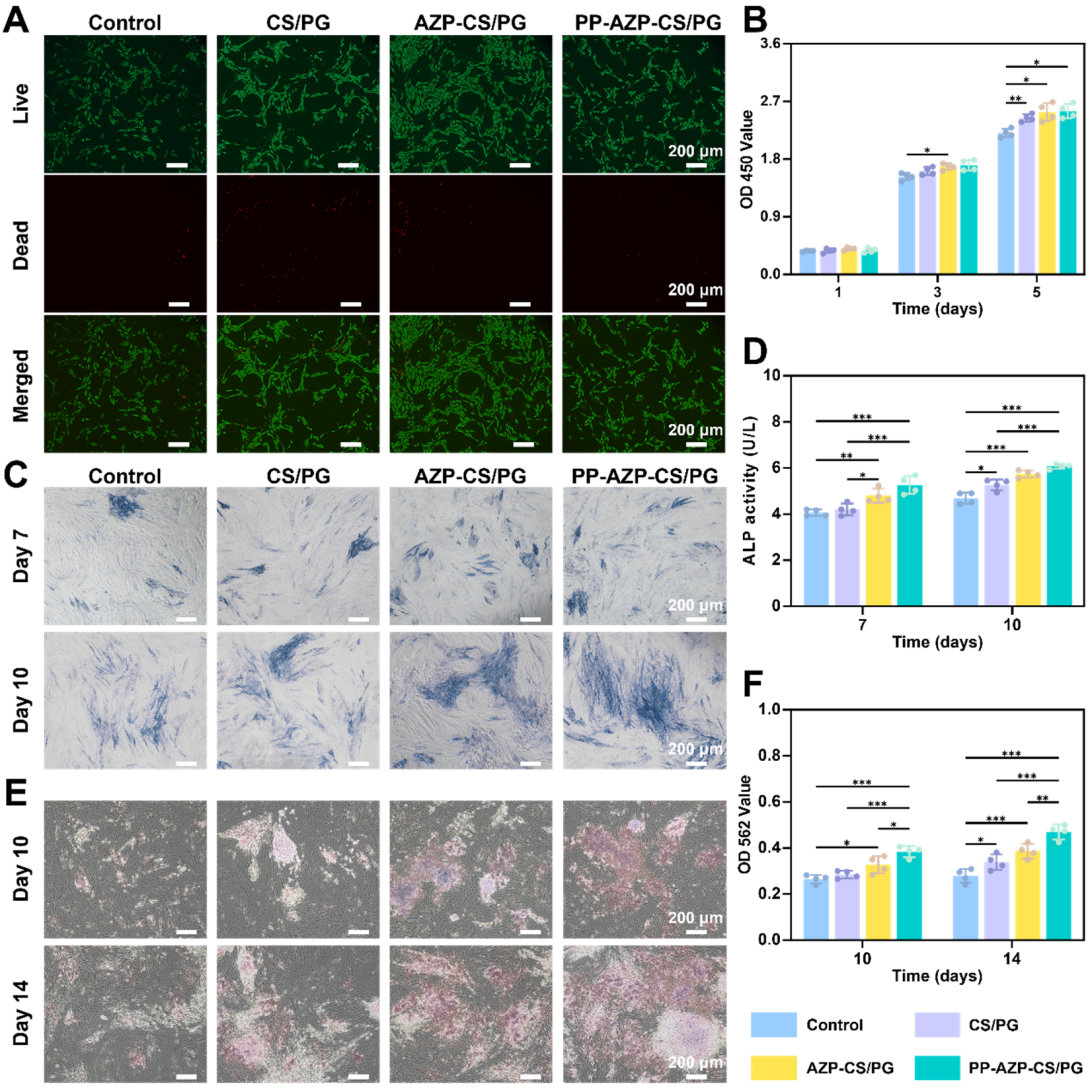
Effects of biomimetic PP-AZP-CS/PG scaffolds on MC3T3-E1 cell proliferation and osteogenic differentiation. (**A**) Live/dead staining of MC3T3-E1 cells at day 1. (**B**) CCK-8 assay of MC3T3-E1 cells at 1, 3, and 5 days. (**C**) Representative ALP staining of MC3T3-E1 cells at 7 and 10 days. (**D**) Quantitative analysis of ALP in MC3T3-E1 cells at 7 and 10 days. (**E**) Representative ARS staining of MC3T3-E1 cells at 10 and 14 days. (**F**) Quantitative analysis of ARS staining in MC3T3-E1 cells at 10 and 14 days. (* *p* < 0.05, ** *p* < 0.01, *** *p* < 0.001).

Given that MC3T3-E1 cells possess the ability to differentiate into osteoblasts and participate in bone repair-related expression, these cells were selected to evaluate the in vitro biocompatibility and osteogenic induction potential of the as-prepared scaffold [45,46]. Live/dead staining (Figure 7A) displayed predominantly green fluorescence with minimal red signal in all groups, indicating no apparent cytotoxicity. The CCK-8 assay (Figure 7B) showed a continuous increase in cell viability over the culture period in all groups. By day 5, the OD values in the AZP-CS/PG and PP-AZP-CS/PG groups reached 2.53 and 2.55, while the value was 2.22 for the control group, indicating that the incorporation of AZP and PP further promotes the sustained proliferation of MC3T3-E1 cells. To further evaluate the osteoinductive capacity of the biomimetic scaffolds, ALP and ARS staining were performed to reflect the expression of early and late osteogenic differentiation [47,48]. ALP is a representative early osteogenic marker that participates in extracellular matrix mineralization. ALP staining showed that the intensity of ALP expression increased with longer culture times (Figure 7C). On day 10, PP-AZP-CS/PG exhibited deeper blue-violet deposits and a larger stained area, followed by the AZP-CS/PG group. The CS/PG and control groups showed relatively weaker staining. Additionally, quantitative ALP activity analysis (Figure 7D) demonstrated that on day 10, the ALP activity levels in PP-AZP-CS/PG and AZP-CS/PG (6.17 and 5.82, respectively) were significantly higher than those in CS/PG (5.26) and the control (4.68). Subsequently, ARS staining was performed to assess mineralized nodule formation as an indicator of late-stage osteogenesis (Figure 7E). With culture extended to day 14, calcium deposition increased in all groups. Notably, PP-AZP-CS/PG exhibited more abundant red mineralized deposits, followed by AZP-CS/PG, whereas the CS/PG and control groups showed relatively limited calcification. Quantitative analysis further confirmed these observations (Figure 7F). On day 14, PP-AZP-CS/PG showed an ARS absorbance of 0.47, which was significantly higher than that of the control (0.28). These results demonstrated that the biomimetic scaffolds exhibited good biocompatibility while further enhancing early osteogenesis and late-stage mineralization, with the PP-AZP-CS/PG group showing the most outstanding performance.

### 3.3. In Vivo Assessment of Bone Regeneration

**Figure 8:**
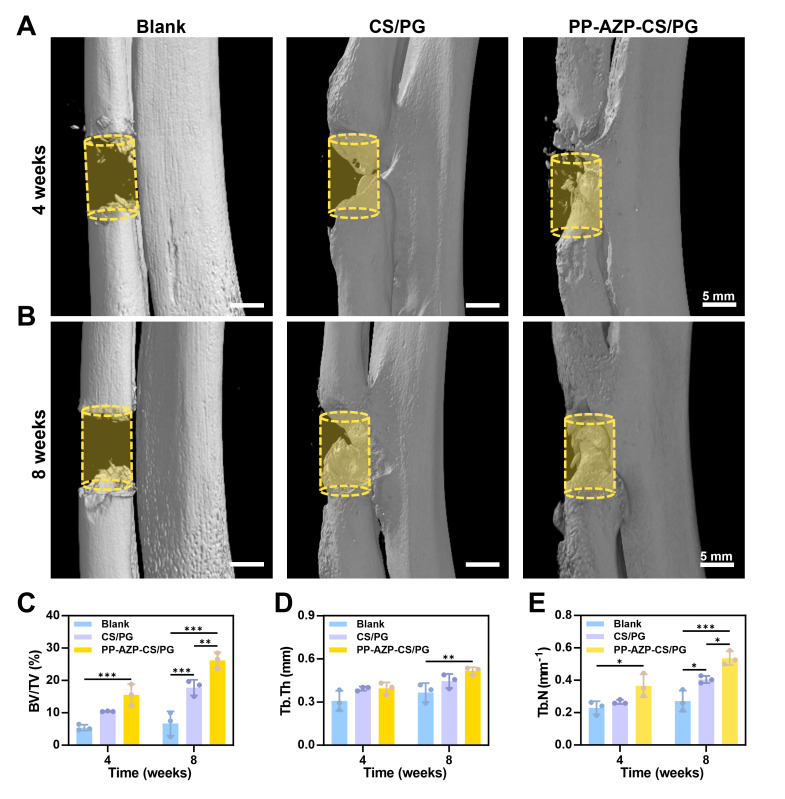
Evaluation of bone regenerative effects of different scaffolds in New Zealand white rabbit radial segmental bone defect model. Representative micro-CT 3D reconstructed images of defect region at 4 weeks (**A**) and 8 weeks (**B**) after scaffold implantation. Yellow scaffold shapes superimposed on images are manually overlaid to highlight scaffold location for better visualization. Quantitative analysis of (**C**) BV/TV, (**D**) Tb.Th, and (**E**) Tb.N for blank, CS/PG and PP-AZP-CS/PG groups. (* *p* < 0.05, ** *p* < 0.01, *** *p* < 0.001).

To systematically evaluate the segmental bone regeneration capacity of the biomimetic scaffolds, a radial bone defect model of Ø 4 mm × 6 mm was established in the midshaft of the New Zealand rabbit radius. Tissue samples were collected for multi-dimensional analysis at the 4-week and 8-week post-implantation time points. Micro-CT scanning and 3D reconstruction analysis revealed significant differences in the repair outcomes between different groups at various time points, with clear time- and group-dependent progression (Figure 8A,B). At 4 weeks post-surgery, the blank group still exhibited a noticeable bone defect, with only a small amount of new bone observed at the radius bone ends. By 8 weeks, a small amount of new bone growth was evident on one side of the radius bone end. In the CS/PG group, bone filling increased significantly, with new bone growing into the scaffold from three directions: both ends of the scaffold and the interface with the ulna. At 4 weeks, new bone growth was visible at both sides of the scaffold, extending toward the center and wrapping around the scaffold. At 8 weeks, clear bone tissue growth into the scaffold was observed on one side, although the middle portion on the radial side of the scaffold had not yet completed cortical bone regeneration. The PP-AZP-CS/PG group demonstrated even more extensive new bone filling and structural continuity. Bone tissue grew from both ends of the scaffold and the ulna side, with a small amount of new bone visible at the middle portion of the radial side at 4 weeks. By 8 weeks, bone tissue had significantly increased compared to 4 weeks, nearly filling the entire scaffold with new bone. The cortical bone growth on the radial side of the scaffold was continuous. To more accurately and comprehensively assess the bone integration effects of each group, quantitative analyses of the new bone volume/total volume ratio (BV/TV), trabecular thickness (Tb.Th), and trabecular number (Tb.N) were performed at 4 weeks and 8 weeks (Figure 8C–E). The results showed that, compared to the control group, both the CS/PG and PP-AZP-CS/PG groups exhibited higher new bone volume, greater trabecular thickness, and increased trabecular number. At 8 weeks, the BV/TV of the PP-AZP-CS/PG group reached 26.22%, far surpassing the 17.78% observed in the CS/PG group and the 6.70% in the blank group. The trabecular thickness in the PP-AZP-CS/PG group (0.52 mm) was also higher than the 0.37 mm in the blank group and the 0.45 mm in the CS/PG group, with no significant difference observed between the PP-AZP-CS/PG and CS/PG groups. The Tb.N in the PP-AZP-CS/PG group reached 0.54 mm^−1^, which was significantly higher than that in the CS/PG group (0.40 mm^−1^) and the blank group (0.27 mm^−1^). Based on the in vitro and in vivo osteogenic assessments, the biomimetic PP-AZP-CS/PG scaffold supported cell proliferation and promoted osteogenic differentiation. Moreover, the porcupine quill-mimetic architecture appeared to facilitate integration with the defect site, likely by enabling early Zn^2+^ release within the oriented foam and by enhancing PP-mediated neurovascular interactions, which together promote neovascularization. Vascular ingrowth improved oxygen and nutrient delivery to the defect site and strengthened angiogenesis–osteogenesis coupling through pathways such as PI3K–Akt and MAPK/ERK, enhancing osteogenic differentiation and matrix mineralization, thereby further supporting the formation of vascularized bone [49,50]. Overall, the PP-AZP-CS/PG scaffold improved trabecular bone ingrowth and enhanced osteointegration.

## 4. Conclusions

In this study, we designed a biomimetic PP-AZP-CS/PG scaffold inspired by porcupine quills via FDM and unidirectional freeze casting to address the limitations in mechanical support and the biological functionality in segmental bone defect. The biomimetic design of a stiff outer PG layer and oriented inner CS foam provided mechanical stability and structural guidance, while the incorporation of PP and AZP within the foam promoted conductive and pro-angiogenic functionality. The in vitro results indicated that the scaffold exhibited excellent biocompatibility while significantly enhancing neovascularization and osteogenic differentiation. Overall, this biomimetic strategy demonstrated translational potential for enhancing clinical performance by synergistically providing mechanical support through structural design and bioactive component stimulation while accelerating the healing of segmental bone defects.

## Data Availability

The original contributions presented in the study are included in the article, further inquiries can be directed to the corresponding authors.

## Supplementary Materials

The following supporting information can be downloaded at https://www.mdpi.com/article/10.3390/jfb17040177/s1. Figure S1: The XRD pattern of natural *Hystrix* quills; Figure S2: In vitro degradation curves of CS foams at different concentrations; Figure S3: Optical images of the capillary absorption of red ink into the biomimetic CS foam. The red hydrophilic ink fully infiltrated the CS foam within 3 s; Figure S4: Stress-strain curves for 2% CS/PG, 3.5% CS/PG, and 5% CS/PG composites; Figure S5: Measurement of the electrical conductivity of CS and 5%PP-CS foam; Table S1: Parameters for FDM printing; Table S2: Compressive strength of PG composites with different etherification parameters at 25% strain.

## Abbreviations

The following abbreviations are used in this manuscript:

ALP	Alkaline phosphatase
ARS	Alizarin red S
AZP	Amorphous zinc phosphate
PP	PEDOT:PSS
PVA	Polyvinyl alcohol
GLY	Glycerol
CS	Chitosan
PG	Polyvinyl alcohol–glycerol
CS/PG	Chitosan/polyvinyl alcohol–glycerol scaffold
AZP-CS/PG	Amorphous zinc phosphate/chitosan/polyvinyl alcohol–glycerol scaffold
PP-AZP-CS/PG	PEDOT:PSS–amorphous zinc phosphate–chitosan/polyvinyl alcohol–glycerol scaffold
BV/TV	Bone volume/total volume
Tb.Th	Trabecular thickness
Tb.N	Trabecular number
FDM	Fused deposition modeling
FBS	Fetal bovine serum
EDS	Energy-dispersive X-ray spectroscopy
FT-IR	Fourier transform infrared spectroscopy
HUVECs	Human umbilical vein endothelial cells
MC3T3-E1	Mouse pre-osteoblast cell line
PAA	Polyacrylic acid
SEM	Scanning electron microscopy
TEM	Transmission electron microscopy
TGA	Thermogravimetric analysis
VEGF	Vascular endothelial growth factor
XRD	X-ray diffraction
