# Supplementary material for: Biomimetic Chitosan/Polyvinyl Alcohol–Glycerol Scaffolds Inspired by Porcupine Quills for Segmental Bone Defect Repair
Source: J Funct Biomater. 2026 Apr 3;17(4):177. doi: 10.3390/jfb17040177 (PMC13118015; doi:10.3390/jfb17040177)
Supplement: Supplementary file 1 [file jfb-17-00177-s001.zip › jfb-4178449-supplementary.pdf]

Supplementary data

Biomimetic Chitosan/Polyvinyl Alcohol-glycerol Scaffolds inspired by Porcupine Quills for Segmental Bone Defect Repair

Jingwen Yang [1,2,†], Zihao Zhao [1,2,†], Zengtao Song [3,4,5], Lei Cao [1,2], Xifan Mei [3,4,5,*] and Xing Zhang [1,2,*]

[1] Institute of Metal Research, Chinese Academy of Sciences, Shenyang 110016, China

[2] School of Materials Science and Engineering, University of Science and Technology of China, Shenyang 110016, China

[3] Liaoning Provincial Key Laboratory of Medical Tissue Engineering, Jinzhou 121000, China

[4] Liaoning Provincial Collaborative Innovation Center of Medical Testing and Drug Development, Jinzhou 121000, China

[5] Liaoning Provincial Collaborative Innovation Center for Health Promotion of Children and Adolescents of Jinzhou Medical University, Jinzhou 121000, China

* Correspondence: meixifan@jzmu.edu.cn (X.M.); xingzhang@imr.ac.cn (X.Z.)

[†] These authors contributed equally to this work.

Supplementary Figure

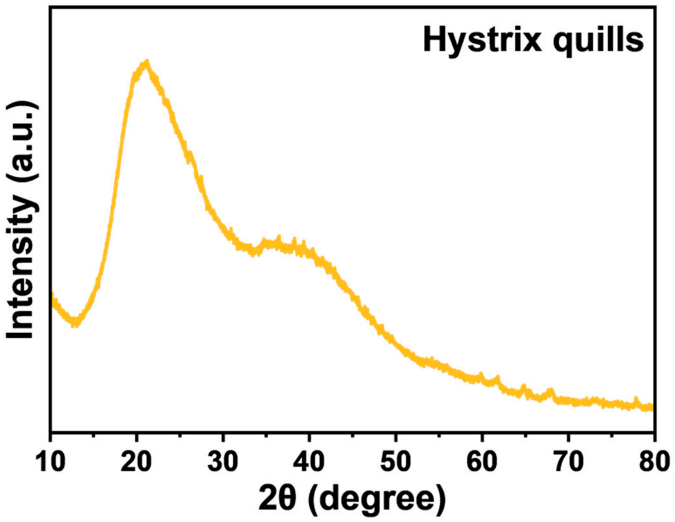

Figure S1. The XRD pattern of natural *Hystrix* quills.

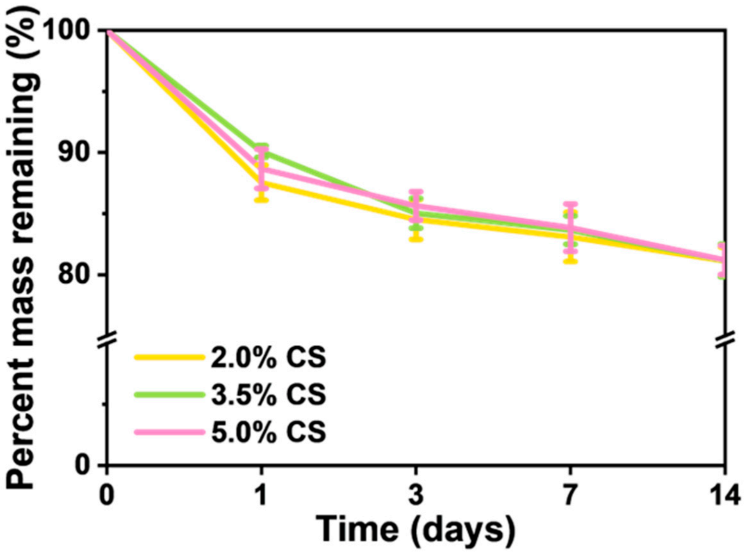

Figure S2. *In vitro* degradation curves of CS foams at different concentrations.

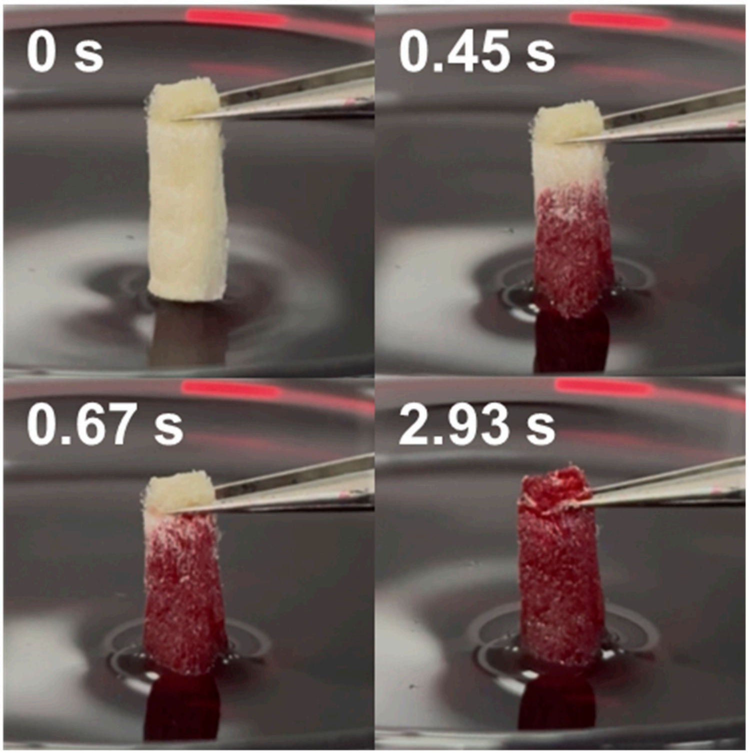

Figure S3. Optical images of the capillary absorption of red ink into the biomimetic CS foam. The red hydrophilic ink fully infiltrated the CS foamwithin 3 seconds.

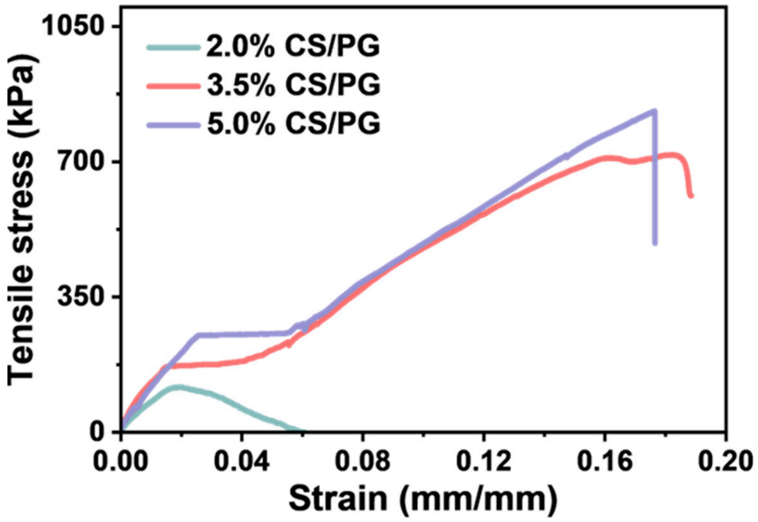

Figure S4. Stress-strain curves for 2% CS/PG, 3.5% CS/PG, and 5% CS/PG composites.

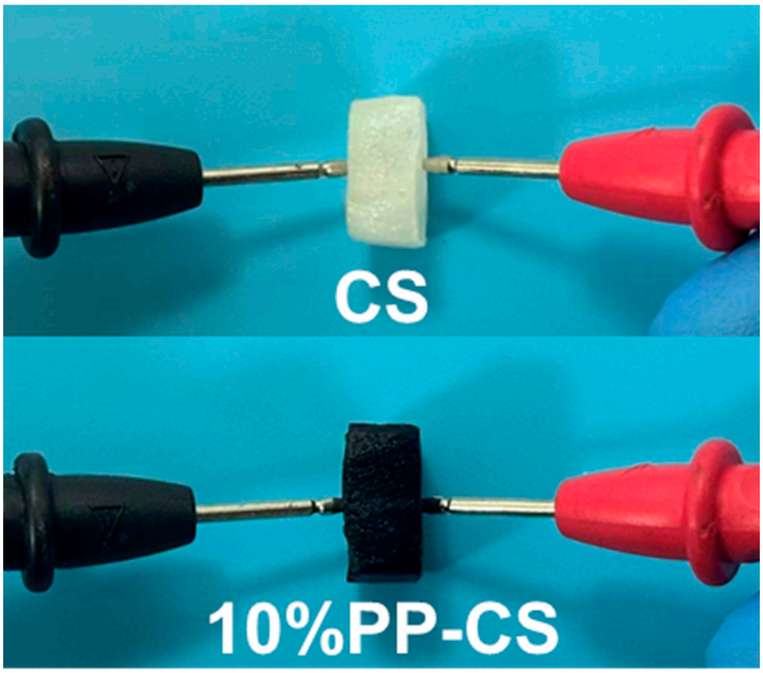

Figure S5. Measurement of the electrical conductivity of CS and 10%PP-CS foam.

Supplementary Table

Table S1. Parameters for FDM printing.

Parameter	Value
Barrel temperature (°C)	260
Layer height (mm)	0.265
Print speed (mm/s)	7
Broken thread elevation (mm)	1
Advance wire feed (ms)	0
Advance wire stop (mm)	1.5
wire feed speed (mm/s)	0.1
Wire feed pre-pressure speed (mm/s)	10
Wire feed pre-pressure time (mm)	200
Wire stop pre-pressure speed (mm/s)	10
Wire stop pre-pressure time (mm)	200

Table S2. Compressive strength of PG composites with different etherification parameters at 25% strain .

	12 hours	24 hours	48 hours
120℃	6.03 ± 0.17 MPa	12.61 ± 2.23 MPa	12.03 ± 0.81 MPa
140℃	14.76 ± 3.07 MPa	24.21 ± 0.11 MPa	26.38 ± 2.44 MPa
160℃	23.87 ± 1.37 MPa	23.83 ± 2.36 MPa	25.06 ± 1.79 MPa
